# A Case Report: The Utility of Multimodality Imaging in the Diagnosis of Cardiac Sarcoidosis–Has It Surpassed the Need for a Biopsy?

**DOI:** 10.3390/reports8010028

**Published:** 2025-03-06

**Authors:** Ali Malik, Paul Ippolito, Sukruth Pradeep Kundur, Sanjay Sivalokanathan

**Affiliations:** 1 Faculty of Life Sciences and Medicine, King’s College London, London WC2R SLS, UK; 2 Department of Cardiology, Icahn School of Medicine at Mount Sinai Morningside, New York, NY 10025, USA

**Keywords:** cardiac sarcoidosis, multimodality imaging, non-invasive diagnosis, endomyocardial biopsy, heart failure, case report

## Abstract

**Background and Clinical Significance:** Cardiac sarcoidosis (CS) is a rare but life-threatening disorder, occurring in 2–5% of sarcoidosis cases, though post-mortem studies suggest a higher prevalence. It presents diagnostic challenges due to nonspecific symptoms and the low sensitivity of an endomyocardial biopsy. Recent guidelines emphasize multimodal imaging, such as cardiac magnetic resonance imaging (MRI) and positron emission tomography (PET). Given the risk of heart failure (HF) and arrhythmias, early detection is critical. This case highlights the role of non-invasive imaging in diagnosing CS and guiding treatment. **Case Presentation:** A 54-year-old female with asthma, hyperlipidemia, a recent diagnosis of anterior uveitis, and familial sarcoidosis presented with dyspnea, chest tightness, and worsening cough. Examination revealed anterior uveitis, erythema nodosum, jugular venous distension, and pedal edema. The electrocardiogram (ECG) demonstrated bifascicular block and premature ventricular contractions (PVCs). The brain natriuretic peptide (BNP) was 975 pg/mL, with the transthoracic echocardiogram revealing a left ventricular ejection fraction of 25–30% with global LV akinesis. Coronary computed tomography angiography (CCTA) excluded coronary artery disease. Cardiac MRI showed late gadolinium enhancement, with PET demonstrating active myocardial inflammation, supporting a >90% probability of CS. Given her clinical trajectory and risk of further decompensation, immunosuppressive therapy was initiated without pursuing a biopsy. A dual-chamber implantable cardioverter defibrillator (ICD) was placed due to risk of ventricular arrhythmias. Bronchoalveolar lavage (BAL) showed a CD4/CD8 ratio of 6.53, reinforcing the diagnosis. She responded well to treatment, with symptom improvement and repeat imaging demonstrating signs of disease remission. **Conclusions:** This case underscores the growing role of multimodal imaging in CS diagnosis, potentially replacing biopsy in select cases. Early imaging-based diagnosis enabled timely immunosuppression and ICD placement, improving outcomes.

## 1. Introduction and Clinical Significance

Cardiac sarcoidosis (CS) poses a significant diagnostic challenge, often being referred to as the great masquerader. It is reportedly only identified in 2–5% of sarcoidosis cases, despite post-mortem studies demonstrating cardiac involvement in 25% of sarcoidosis patients [1]. The wide range of nonspecific clinical presentations results in the diagnosis of cardiac sarcoidosis as a challenge. Four distinct guidelines for diagnosing cardiac sarcoidosis have been proposed by the Japanese Ministry of Health and Welfare (MHLW), the World Association of Sarcoidosis and Other Granulomatous Disorders (WASOG), the Heart Rhythm Society (HRS), and most recently, the Japanese Circulation Society (JCS) [2]. While these guidelines traditionally require histological confirmation of non-caseating granulomas, the low sensitivity of endomyocardial biopsy due to the patchy inflammatory distribution significantly limits its utility [3]. Notably, the latest JCS guidelines emphasize the role of clinical signs and multimodal imaging, such as cardiac magnetic resonance imaging (MRI) and positron emission tomography (PET), acknowledging MRI and PET’s growing evidence for use in detecting inflammation and disease activity [4].

Heart failure due to CS is associated with poor long-term outcomes, with 10-year survival rates ranging from 19–53%, underscoring the urgent need for early and accurate diagnosis [5]. This emphasizes the importance of a strong, reliable diagnostic method that enables prompt and effective treatment initiation. We present a case of new-onset heart failure secondary to CS in which multimodality imaging was utilized to diagnose the disorder and guide prompt medical intervention, thereby highlighting a potential shift towards noninvasive methods in diagnosing cardiac sarcoidosis.

## 2. Case Presentation

**Figure 1:**
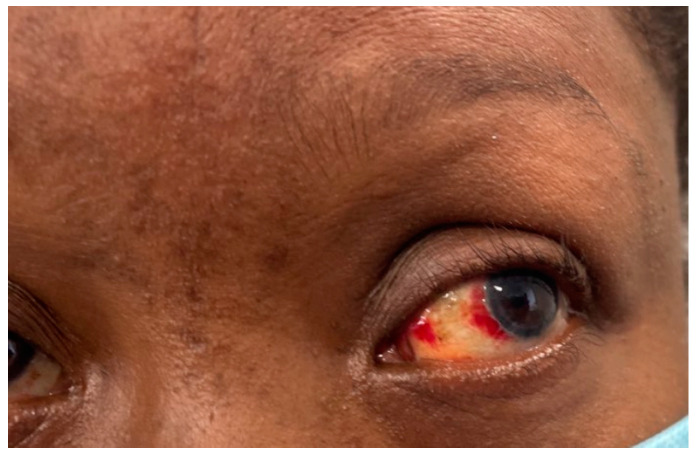
Eye examination demonstrating anterior uveitis.

A 54-year-old female with a significant medical history of asthma, hyperlipidemia, and a recent diagnosis of anterior uveitis presented to the emergency department initially with progressive dyspnea and chest tightness while lying flat. Clinical observations included a heart rate of 76, blood pressure of 140/70 mmHg, and oxygen saturation of 96%. On physical examination, the patient demonstrated a lower extremity rash in line with erythema nodosum and an eye examination that confirmed active anterior uveitis (Figure 1). More importantly, there was jugular venous distension with mild pedal edema.

### 2.1. Investigations

**Figure 2:**
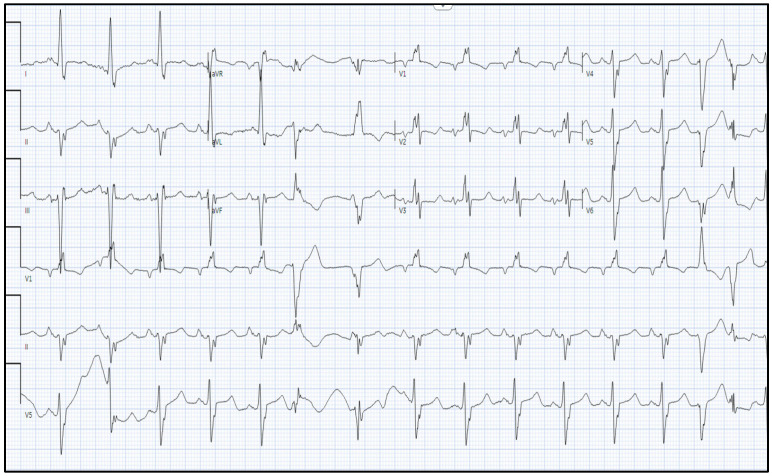
Electrocardiogram demonstrating right bundle branch block, left anterior fascicular block, with frequent premature ventricular contractions and a prolonged QT.

**Figure 3:**
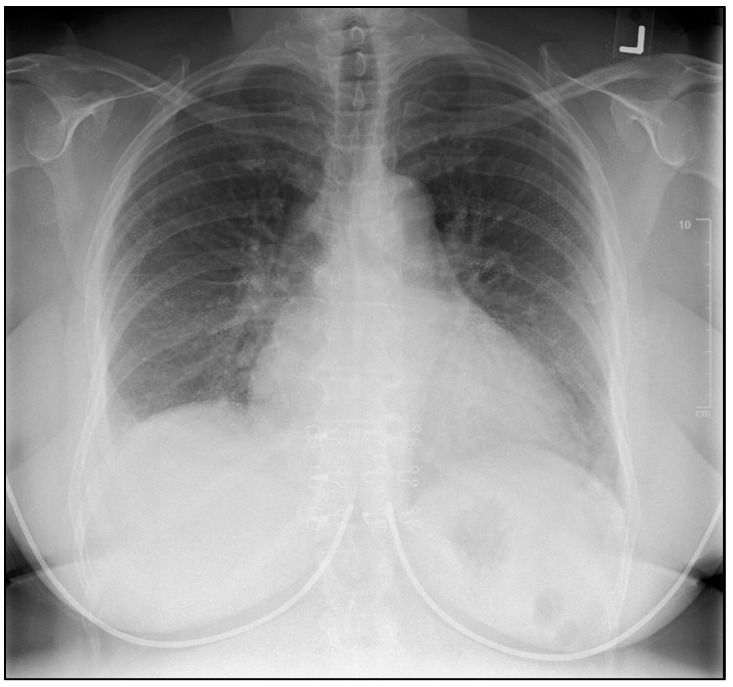
Chest X-ray demonstrating cardiomegaly and pulmonary congestion.

Initial investigations revealed a B-type natriuretic peptide (BNP) level of 975, an electrocardiogram (ECG) showing bifascicular block [right bundle branch block and left anterior fascicular branch (LAFB)], with frequent premature ventricular complexes (PVCs) (Figure 2). An initial chest x-ray demonstrated a degree of cardiomegaly with mild pulmonary congestion (Figure 3). The patient was subsequently admitted for possible new onset heart failure (HF). Transthoracic echocardiography (TTE) demonstrated a newly reduced left ventricular ejection fraction (LVEF) of 25–30%, with left ventricular akinesis and dilation of both the left atrium and right ventricle. Coronary computed tomography angiography (CCTA) excluded coronary artery disease. Subsequently, the reduced LVEF was attributed to non-ischemic cardiomyopathy. Notably, the suspicion of CS was high given the presence of systemic symptoms, as aforementioned, in combination with a strong family history (three first cousins with sarcoidosis). Differential diagnoses included myocarditis, amyloidosis, or other forms of infiltrative cardiomyopathies. After stabilization of her acute HF presentation, the patient was commenced on guideline-directed medical therapy (GDMT), including metoprolol 50 mg, valsartan 20 mg, empagliflozin 10 mg, and spironolactone 12.5 mg.

**Figure 4:**
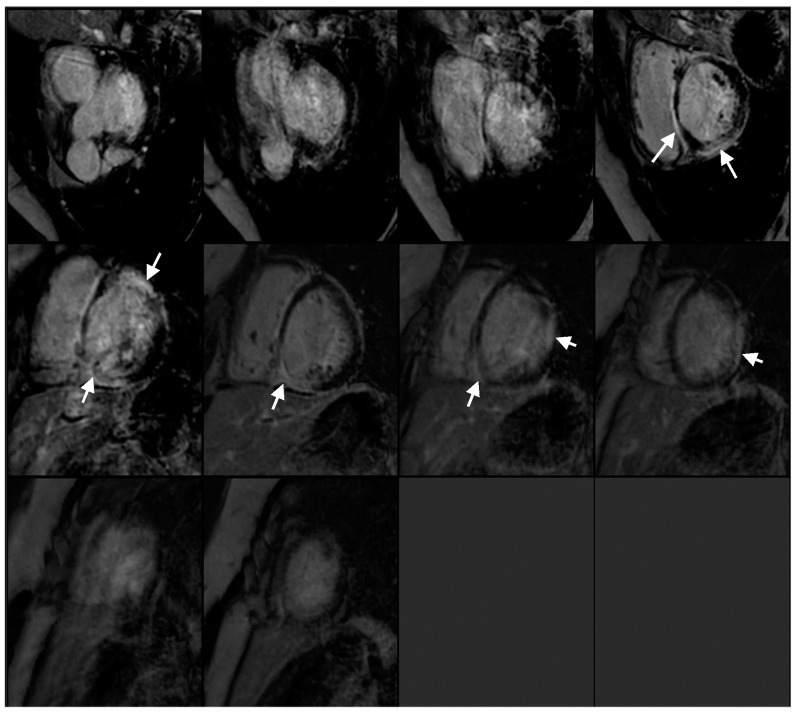
Cardiac MRI with multifocal LGE (arrows) consistent with an inflammatory cardiomyopathy.

**Figure 5:**
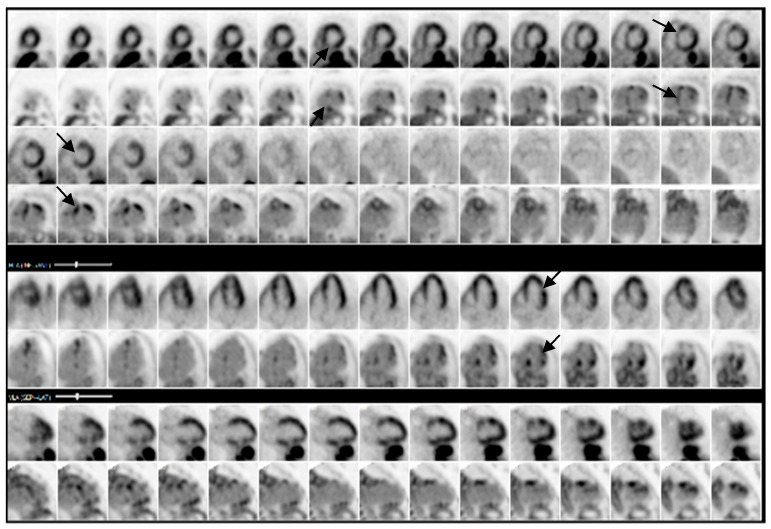
PET demonstrating multi-focal FDG uptake (arrows) with associated matched perfusion abnormalities in the basal and mid-inferior and inferoseptal segments, basal anterior, basal to mid-anterolateral segments, and apical lateral and inferior segments.

**Figure 6:**
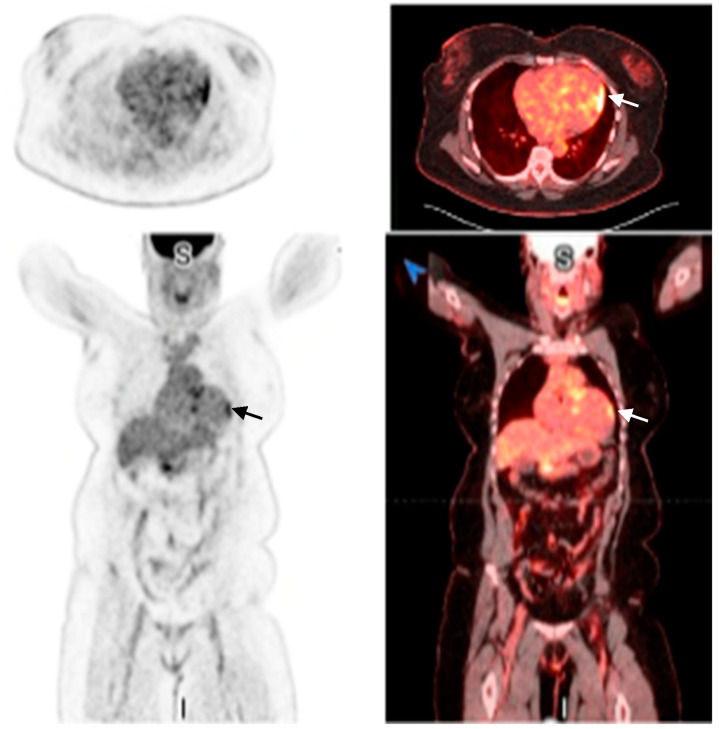
FDG PET-CT showing myocardial FDG uptake (arrows).

The cardiac MRI revealed multifocal and heterogeneous late gadolinium enhancement (LGE) affecting the basal to mid-interventricular wall, specifically within the anteroseptal and inferoseptal regions. Furthermore, there was evidence of transmural LGE in the mid-anterior, mid-lateral, and mid-inferior segments (Video S1, Figure 4). PET revealed a mismatch pattern with active cardiac inflammation, indicating a >90% probability of cardiac sarcoidosis, supporting our clinical suspicion (Figure 5 and Figure 6). Given these findings, a treatment plan was devised to manage both cardiac and systemic manifestations of sarcoidosis.

### 2.2. Management

Given the recent diagnosis of severe heart failure (≤35%), there was a concern regarding the potential rapid progression of sarcoidosis. Therefore, the patient underwent placement of a dual-chamber implantable cardioverter-defibrillator (ICD). Immunosuppressive therapy was initiated without confirmation from a biopsy, given the severity and urgency of her presentation. Methotrexate (MTX) was initiated at a dosage of 15 mg weekly, titrated over six weeks, in conjunction with prednisone, which was prescribed at a dosage of 30 mg daily and subsequently tapered to 5 mg daily over a 12-week period. Furthermore, pantoprazole was administered for gastrointestinal protection, and ivermectin was provided for the prophylaxis of strongyloidiasis. It was determined that *Pneumocystis jirovecii* pneumonia (PJP) prophylaxis would be deferred, considering the expedited tapering of corticosteroids. Bronchoscopy with bronchoalveolar lavage (BAL) and cytology revealed a CD4/CD8 ratio of 6.53, further supporting a diagnosis of sarcoidosis. Unfortunately, no associated lymph node was amenable to being biopsied. Flow cytometry demonstrated an 89.1% population of CD3+ T lymphocytes, with a predominance of CD4+ cells, further solidifying the diagnosis of sarcoidosis.

**Figure 7:**
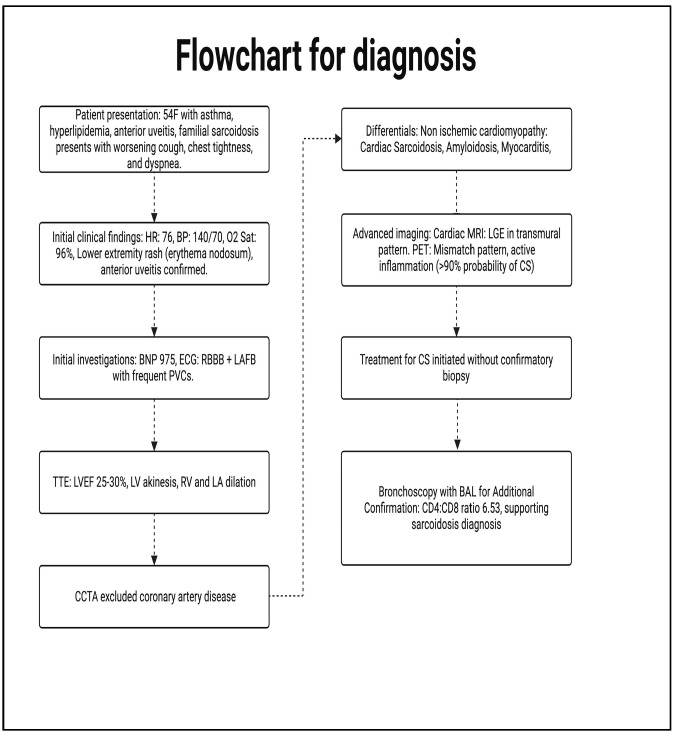
Flowchart highlighting our stepwise approach to diagnosing CS in this case.

At the follow-up visit (Table 1, Figure 7), the patient reported significant improvement in heart failure symptoms, including reduced dyspnea and fatigue. Repeat TTE and PET revealed hemodynamic improvement and signs of disease remission, respectively. Unfortunately, during her ICD interrogation, a brief episode of paroxysmal atrial fibrillation, lasting four minutes, was discovered. Given a CHA_2_DS_2_-VASc score of 2 (female, HF), the patient was started on apixaban.

**Table 1:** Timeline of Presentation.

Date	Event
4 September 2024	Patient presents to the emergency department - Started on GDMT for HFrEF
13 September 2024	PET shows >90% probability of cardiac sarcoidosis
14 September 2024	ICD implantation - Commencement of immunosuppressive therapy
22 October 2024	Bronchioalveolar lavage supports diagnosis of sarcoidosis
25 November 2024	Brief atrial fibrillation episode noted: initiated on apixaban
Present	Patient reports improvement in HF symptoms - Follow-up TTE and PET demonstrating signs of improvement

## 3. Discussion

This case highlights an acute presentation of cardiac sarcoidosis, highlighting the critical role of multimodal imaging techniques—such as CT, MRI, and PET—in facilitating a timely and accurate diagnosis. This is particularly significant given the patient’s substantial impairment in left ventricular function and elevated risk of ventricular arrhythmias. A biopsy was strongly considered; however, there were no suitable targets. The decision to commence immunosuppressive therapy prior to obtaining histopathological confirmation through biopsy was deemed appropriate due to the strong clinical suspicion, high-risk clinical presentation, and the need to circumvent potential complications associated with biopsy procedures. Nevertheless, it is important to acknowledge that this strategy has inherent limitations, as histopathological confirmation is regarded as the gold standard for diagnosing cardiac sarcoidosis [6].

CS is a rare but potentially life-threatening condition, often presenting with heart failure, arrhythmias, or conduction abnormalities [7]. The presence of bifascicular block and PVCs on ECG, may be an early indicator of cardiac involvement, and can be found in CS patients [8]. Cardiac MRI is a highly sensitive modality for detecting inflammatory cardiomyopathy [9]. In case of sarcoidosis with cardiac involvement, LGE is predominantly observed in the basal inferolateral segments, followed by basal anteroseptal and basal inferoseptal segments. Other common locations include mid-anteroseptal, mid-inferoseptal, mid-inferior and mid-inferolateral segments [10]. More importantly, PET further supplements the diagnosis by demonstrating active inflammation, a key feature that guides the decision to initiate immunosuppression [11]. There is concordance between segmental uptake of FDG on PET and LGE on cardiac MRI, with greatest concordance being noted in the basal anteroseptal and mid inferoseptal regions [10].

This case report questions whether multimodal imaging has surpassed the need for biopsy in diagnosing CS, providing compelling evidence that in specific high-probability scenarios, it may have. Cardiac MRI and PET have emerged as essential tools, offering high sensitivity and specificity for detecting active inflammation. While biopsy remains the gold standard, it is limited by procedural risks and sampling errors, often yielding false-negative results due to patchy myocardial involvement. The latest literature on endomyocardial biopsy reports its sensitivity to be as low as <25% [12]. In this case report, the amalgamation of clinical presentation, imaging findings, and systemic sarcoidosis manifestations enabled a confident diagnosis without the need for tissue confirmation, allowing for early intervention and improving patient outcomes.

The existing literature on acute CS extensively supports the early placement of ICD in patients diagnosed with heart failure with reduced ejection fraction (HFrEF), given the heightened risk of sudden cardiac death in this population [13]. The decision to initiate treatment with steroids and methotrexate is consistent with current clinical guidelines for the management of active inflammation associated with CS [14]. Considering the systemic nature of sarcoidosis, an interdisciplinary approach was imperative, necessitating collaboration among specialists in cardiology, pulmonology, ophthalmology, and rheumatology to provide comprehensive and effective care.

## 4. Conclusions

This case emphasizes the utility of non-invasive techniques in diagnosing CS, a frequently overlooked cause of heart failure. More importantly, it raises the question regarding the necessity of biopsy in cases with a high probability of diagnosis, facilitating early immunosuppressive therapy and timely management of the risk of ventricular arrhythmias.

## Data Availability

The original contributions presented in this study are included in the article/Supplementary Materials. Further inquiries can be directed to the corresponding author.

## Supplementary Materials

The following supporting information can be downloaded at: https://www.mdpi.com/article/10.3390/reports8010028/s1, Video S1: Cardiac MRI demonstrating biventricular dysfunction.

## Abbreviations

BAL	Bronchoalveolar Lavage
BNP	B-type Natriuretic Peptide
CCTA	Coronary Computed Tomography Angiography
CS	Cardiac Sarcoidosis
ECG	Electrocardiogram
GDMT	Guideline-Directed Medical Therapy
HF	Heart Failure
HFrEF	Heart Failure with Reduced Ejection Fraction
ICD	Implantable Cardioverter-Defibrillator
JCS	Japanese Circulation Society
LAFB	Left Anterior Fascicular Block
LGE	Late Gadolinium Enhancement
LV	Left Ventricle
LVEF	Left Ventricular Ejection Fraction
MRI	Magnetic Resonance Imaging
MTX	Methotrexate
PJP	*Pneumocystis Jirovecii* Pneumonia
PVCs	Premature Ventricular Complexes
PET	Positron Emission Tomography
RBB	Right Bundle Branch
TTE	Transthoracic Echocardiography
WASOG	World Association of Sarcoidosis and Other Granulomatous Disorders
